# Dyspnea and the Varying Pathophysiologic Manifestations of Chronic Obstructive Pulmonary Disease Evaluated by Cardiopulmonary Exercise Testing With Arterial Blood Analysis

**DOI:** 10.3389/fphys.2018.01293

**Published:** 2018-10-02

**Authors:** Hiroyuki Kagawa, Keisuke Miki, Seigo Kitada, Mari Miki, Kenji Yoshimura, Yohei Oshitani, Kohei Nishida, Nobuhiko Sawa, Kazuyuki Tsujino, Ryoji Maekura

**Affiliations:** ^1^Department of Respiratory Medicine, National Hospital Organization Toneyama National Hospital, Toyonaka,Japan; ^2^Graduate School of Health Care Sciences, Jikei Institute, Osaka, Japan

**Keywords:** dyspnea, exercise tolerance, hypoxemia, acidosis, norepinephrine, COPD, cardiopulmonary exercise testing

## Abstract

**Background:** Patients with chronic obstructive pulmonary disease (COPD) show varying mechanisms of exertional dyspnea with different exercise capacities.

**Methods:** To investigate the pathophysiologic conditions related to exertional dyspnea, 294 COPD patients were evaluated using cardiopulmonary exercise testing (CPET) with arterial blood analyses, with the patients classified into two groups according to their exercise limitation: the leg fatigue group (*n* = 58) and the dyspnea group (*n* = 215). The dyspnea group was further subdivided into four groups based on peak oxygen uptake (${\overset{{}^{\circ}}{\text{V}}}_{\text{O}2}$ in mL/min/kg): group A (< 11), group B (11 to < 15), group C (15 to < 21), and group D (≥21).

**Results:** In the dyspnea group, group A (n = 28) showed the following findings: (i) the forced expiratory volume in 1 s was not correlated with the peak ${\overset{{}^{\circ}}{\text{V}}}_{\text{O}2}$ (*p* = 0.288), (ii) the arterial oxygen tension (PaO_2_) slope (peak minus resting PaO_2_/Δ${\overset{{}^{\circ}}{\text{V}}}_{\text{O}2}$) was the steepest (*p* < 0.0001) among all subgroups, (iii) reduced tidal volume (V_T_) was negatively correlated with respiratory frequency at peak exercise (*p* < 0.0001), and (iv) a break point in exertional V_T_ curve was determined in 17 (61%) patients in group A. In these patients, there was a significant negative correlation between bicarbonate ion (${\text{HCO}}_{3}^{-}$) levels at peak exercise and V_T_ level when the V_T_-break point occurred (*p* = 0.032). In group D (*n* = 46), ${\text{HCO}}_{3}^{-}$ levels were negatively correlated with plasma lactate levels (*p* < 0.0001). In all subgroups, the ${\text{HCO}}_{3}^{-}$ level was negatively correlated with minute ventilation. The dyspnea subgroups showed no significant differences in the overall mean pH [7.363 (SD 0.039)] and Borg scale scores [7.4 (SD, 2.3)] at peak exercise.

**Conclusions:** During exercise, ventilation is stimulated to avoid arterial blood acidosis and hypoxemia, but ventilatory stimulation is restricted in the setting of reduced respiratory system ability. These conditions provoke the exertional dyspnea in COPD. Although symptom levels were similar, the exertional pathophysiologic conditions differed according to residual exercise performance; moreover, COPD patients showed great inter-individual variability. An adequate understanding of individual pathophysiologic conditions using CPET is essential for proper management of COPD patients.

## Introduction

Exertional dyspnea is a major symptom that limits exercise ability in patients with chronic obstructive pulmonary disease (COPD), especially in the advanced stages of the disease (O'Donnell et al., 2009; Parshall et al., 2012). The factors causing exertional dyspnea include single or combined ventilatory disorders secondary to reduced ventilatory capacity, increased ventilatory requirement, and gas exchange abnormalities, including functional skeletal muscle disorder and cardiac dysfunction (Palange et al., 2007). Consequently, exertional dyspnea due to COPD might influence the entire body and make daily living laborious (Laveneziana and Palange, 2012). Therefore, an adequate understanding of an individual patient's condition via evaluation of exercise intolerance and cardiopulmonary function is vital for development of a personalized treatment strategy based on pathophysiology. Using cardiopulmonary exercise testing (CPET), previous studies have reported that (i) COPD patients with severely reduced exercise tolerance develop exercise-induced hypoxemia, sympathetic overactivity, and progressive exertional acidosis at low-intensity exercise (Maekura et al., 2014); (ii) such life-threatening factors are predictors of mortality (Yoshimura et al., 2014); and (iii) screening and avoiding such factors and a pulmonary rehabilitation program with exercise training and occupational therapy can improve the prognosis of patients with severe COPD (Maekura et al., 2015).

Although the disease severity of COPD is based on the degree of airflow limitation, as defined by forced expiratory volume in 1 s (FEV_1_), a few interesting studies have attempted evaluation of COPD severity on the basis of exercise tolerance (Oga et al., 2005; Maekura et al., 2014). Therefore, after grouping patients according to the exercise tolerance determined using CPET and combining those with a similar degree of airflow limitation (FEV_1_), we investigated the pathophysiologic conditions related to exertional dyspnea in a broader spectrum of COPD patients. First, we compared the pathologic conditions between two groups classified according to the reason for exercise limitation during CPET: the dyspnea group and the leg fatigue group. Second, focusing on the dyspnea group, we aimed to investigate (i) whether exercise intolerance was correlated with the degree of air flow limitation and breathing pattern during exercise; (ii) how exercise-induced hypoxemia was influenced by other pathophysiologic variables during exercise; and (iii) the underlying mechanism of exercise-induced acidosis, which often occurs at the end stage of CPET. Considering these aspects, we performed a detailed re-evaluation of the variability of pathophysiological conditions in patients with COPD. We addressed the necessity and usefulness of CPET with arterial blood analysis for evaluation of pathophysiological conditions and management in patients with COPD.

## Materials and methods

### Patients

CPET with arterial blood analyses was consecutively performed in 2,831 patients with exertional dyspnea, based on a modified Medical Research Council score (American Thoracic Society, 1995a; Pauwels et al., 2001; Global Initiative for Chronic Obstructive Lung Disease. GOLD, 2017) of 1 or higher, during clinical practice at our institution between May 1999 and January 2011. We excluded patients who had absolute contraindications to clinical exercise testing (Clinical exercise testing with reference to lung diseases: indications, standardization and interpretation strategies, 1997), who showed COPD exacerbation (respiratory infection) within the past 2 months, and who were participating in a pulmonary rehabilitation program. We selected potential study participants who had been prescribed an appropriate medication for 2 months and underwent CPET for assessment of their pathophysiological conditions before introduction of the pulmonary rehabilitation program. The diagnosis of COPD was confirmed on the basis of the Global Initiative for Chronic Obstructive Lung Disease definition and classification (American Thoracic Society, 1995a; Pauwels et al., 2001). We excluded patients with comorbidities (e.g., severe cardiovascular disease, active tuberculosis, definite sequelae of tuberculosis, asthma, pulmonary fibrosis, or neuromuscular disease) that could contribute to dyspnea and exercise limitation. Thus, we included 294 patients with stable COPD in the study. Although CPET is covered by the Japanese healthcare insurance system, the protocols were fully explained to the participating patients by the attending physicians. All patients provided written informed consent to undergo the protocols before CPET. The protocol of the retrospective study was approved by the institutional review board of the National Hospital Organization Toneyama National Hospital (approval number: 2007-0711) and was in accordance with the Declaration of Helsinki for experiments involving human subjects.

### Cardiopulmonary exercise testing

CPET was performed on a treadmill (Marquette CASE series T 2001; GE Healthcare, Tokyo, Japan). Symptom-limited exercise tests were conducted using the Sheffield protocol or one of the two modified Sheffield protocols after Allen's test, as described previously (Maekura et al., 2014). The exercise protocol was selected on the basis of the patient's daily activities and pulmonary function test results. Progressive incremental exercise testing was discontinued when the subject experienced breathlessness and/or leg fatigue, reached the predicted maximum heart rate (HR), or showed notable electrocardiographic changes, such as an ST segment depression of >2 mm or a short run of premature ventricular contractions. To obtain reliable data, CPET was performed without encouragement, especially during exercise. Pre-exercise resting measurements were obtained during the steady-state period after at least 3 min of breathing through a mask. Data for the expired gas were measured using the Aero monitor AE310S (Minato Medical Science Co., Ltd, Osaka, Japan). Ventilatory values were measured on a breath-by-breath basis and were presented as 30-s averages at rest, at 1- and 3-min intervals during exercise, and at the end of exercise. Dyspnea was measured with the Borg scale (Borg, 1982). Before testing, the Borg scale was explained to the patients and its endpoints were ranked from 0 for “no difficulty in breathing” to 10 for “the most severe difficulty in breathing,” based on the subject's previous experience or perception. The subjects rated their dyspnea at rest, every minute during exercise, and at peak exercise. Immediately after exercise cessation and completion of mechanical measurements, the subjects were asked for their reason(s) for exercise termination (i.e., dyspnea, leg fatigue, both, or others). Arterial blood samples for blood gas analyses and plasma lactate and plasma norepinephrine assessments were collected at rest, during the last 15 s of each exercise stage, and at the end of exercise, as previously described (Maekura et al., 2014). Blood gas analyses were performed using ABL800 FLEX (Radiometer, Copenhagen, Denmark). Indirect maximum voluntary ventilation (MVV) was calculated as FEV_1_ × 35 (Johnson et al., 1999). The dyspnea index was calculated as peak minute ventilation (${\overset{{}^{\circ}}{\text{V}}}_{\text{E}}$)/indirect MVV (Hallstrand et al., 2000). Breathing reserve was calculated as indirect MVV–${\overset{{}^{\circ}}{\text{V}}}_{\text{E}}$ at peak exercise (Wasserman et al., 2005). Predicted maximum HR was calculated as 220–age in years (Wasserman et al., 2005). The percentage of HR reserve was calculated as HR at peak exercise/predicted maximum HR. The change in arterial carbon dioxide tension (ΔPaCO_2_) was calculated as peak PaCO_2_-resting PaCO_2_. The change in oxygen uptake (Δ${\overset{{}^{\circ}}{\text{V}}}_{\text{O}2}$) was calculated as peak ${\overset{{}^{\circ}}{\text{V}}}_{\text{O}2}$-resting ${\overset{{}^{\circ}}{\text{V}}}_{\text{O}2.}$ As a measure of the severity of exercise-induced hypoxemia, arterial oxygen tension (PaO_2_)-slope was calculated as (peak PaO_2_-resting PaO_2_)/Δ${\overset{{}^{\circ}}{\text{V}}}_{\text{O}2}$ (Hiraga et al., 2003). The break points in V_T_, dyspnea (Borg scale), and plasma lactate level during exercise were determined for each subject using the intersection of two lines on individual plots of each parameter's curve (Miki et al., 2009).

### Pulmonary function testing

Post-bronchodilator spirometry (CHESTAC 8800; CHEST M.I. Inc., Tokyo, Japan) was performed. The highest measurements were used for subsequent analyses, as previously described (American Thoracic Society, 1995b; Borrill et al., 2005).

### Data analysis

**Figure 1:**
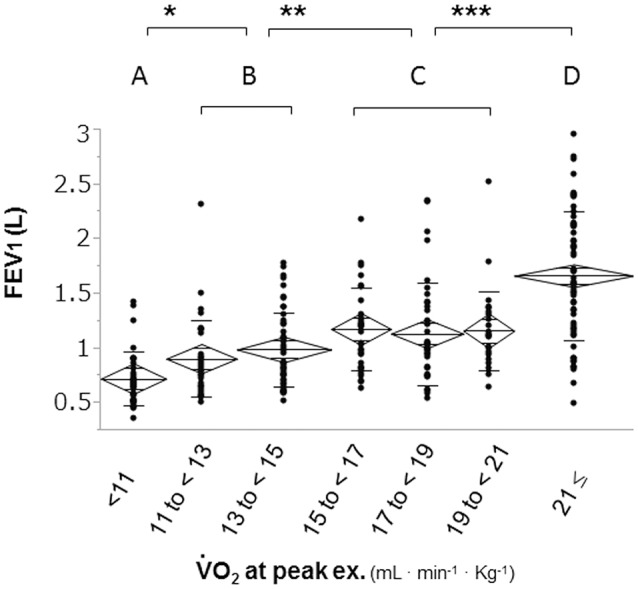
Distribution of FEV_1_ based on exercise tolerance. Patients were divided into seven groups according to increments of 2 mL·min^−1^·kg^−1^ in peak ${\overset{{}^{\circ}}{\text{V}}}_{\text{O}2}$ to confirm the distribution of FEV_1_ at different levels of exercise tolerance. The second subdivision of the patients was into four subgroups: group A (< 11 ml·min^−1^·kg^−1^); group B (11 to < 15 mL·min^−1^·kg^−1^); group C (15 to < 21 mL·min^−1^·kg^−1^), and group D (≥21 mL·min^−1^·kg^−1^). FEV_1_: forced expiratory volume in one second; ${\overset{{}^{\circ}}{\text{V}}}_{\text{O}2}$: oxygen uptake; ^*^p < 0.05, ^**^p < 0.01 ^***^p < 0.0001 using Tukey–Kramer honestly significant test.

**Figure 2:**
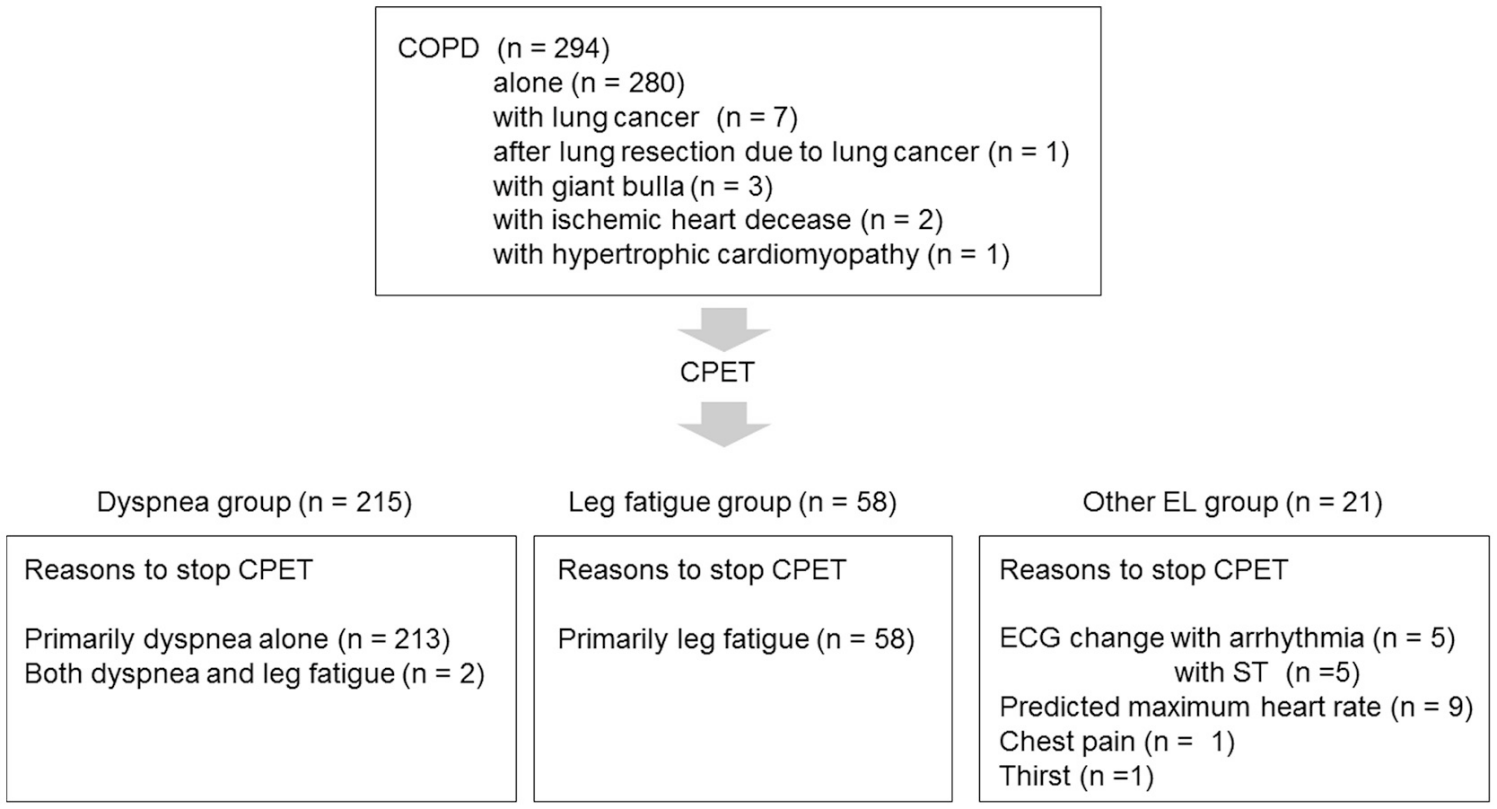
Patient selection flowchart COPD, chronic obstructive pulmonary disease; CPET, cardiopulmonary exercise testing; ECG, electrocardiogram; EL, exercise limitation.

To confirm the distribution of FEV_1_ at different exercise tolerance levels, all patients were grouped into seven groups according to peak ${\overset{{}^{\circ}}{\text{V}}}_{\text{O}2}$ increments of 2 mL·min^−1^·kg^−1^ and into four groups according to similarities in the mean FEV_1_ level (Figure 1). Next, all patients (*n* = 294) were divided into three groups: the dyspnea group (*n* = 215) consisted of patients whose exercise limitation during CPET was primarily due to exertional dyspnea, which was defined as breathing discomfort alone or in conjunction with leg fatigue; the leg fatigue group (*n* = 58) consisted of patients whose exercise limitation was primarily due to leg fatigue; and the other exercise limitations (EL) group (*n* = 21) consisted of patients whose exercise limitation was due to electrocardiogram (ECG) changes, achievement of the predicted maximum HR, chest pain, and thirst (Figure 2).

Statistical analyses were performed using the JMP 11 (SAS Institute Inc., Cary, NY, USA). The reported values were consistently expressed as mean ± standard deviation. Differences between the dyspnea and leg fatigue groups were analyzed using the Student's *t*-test and chi-squared test. A parametric one-way analysis of variance for normally distributed variables was used, and a non-parametric Kruskal–Wallis test for non-normally distributed variables was used to determine the differences in physiologic parameters among the four subgroups in the dyspnea group. Differences between pairs of groups among the dyspnea groups were analyzed using the Tukey–Kramer honestly significant difference test. The relationships between the values obtained by the pulmonary function test and CPET were assessed using the Pearson's correlation coefficient and linear regression analysis. Differences between the groups were considered statistically significant when p values were less than 0.05.

## Results

This study included a total of 294 patients (273 men) with stable COPD; the other characteristics are shown in Table 1.

**Table 1:** Baseline characteristics of the study population.

	**Total (*n* = 294)**	**Dyspnea group (*n* = 215)**	**Leg fatigue group (*n* = 58)**	***p*-value**
Age, years	70.7 (7.4)	70.1 (7.3)	71.7 (7.2)	0.127
BMI, kg·m^−2^	20.6 (3.2)	20.5 (3.1)	20.4 (3.5)	0.850
GOLD, I/II/III/IV	14/81/135/64	7/49/108/51	3/19/24/12	0.250
**PULMONARY FUNCTION TEST**				
FEV_1_, L	1.16 (0.52)	1.10 (0.50)	1.23 (0.54)	0.077
%FEV_1_, % predicted	44.6 (17.7)	42.3 (16.7)	46.8 (17.8)	0.073
FEV_1_/FVC, %	44.4 (11.9)	43.1 (11.6)	45.6 (11.5)	0.151
VC, L	2.89 (0.76)	2.87 (0.77)	2.90 (0.76)	0.832
**CARDIOPULMONARY EXERCISE TESTING**				
At Rest			
PaO_2_, mmHg	81.0 (11.4)	80.2 (11.2)	84.0 (11.8)	0.024
PaCO_2_, mmHg	37.6 (4.7)	37.6 (4.8)	37.7 (4.1)	0.792
pH	7.423 (0.026)	7.423 (0.025)	7.424 (0.004)	0.617
At Peak Exercise
Dyspnea, Borg scale	7.0 (2.5)	7.4 (2.3)	5.8 (0.3)	< 0.0001
Dyspnea index, %	102.3 (24.9)	105.6 (24.9)	96.0 (24.0)	0.009
Breathing reserve, L·min^−1^	1.5 (10.7)	0.0 (10.2)	4.3 (10.6)	0.005
%HR reserve, %	83.9 (13.5)	83.0 (13.8)	83.3 (9.4)	0.856
${\overset{{}^{\circ}}{\text{V}}}_{\text{O}2}$, mL·min^−1^·kg^−1^	16.9 (5.4)	16.9 (5.5)	16.5 (4.9)	0.626
${\overset{{}^{\circ}}{\text{V}}}_{\text{E}}$, L·min^−1^	39.0 (14.2)	38.5 (14.1)	38.9 (13.9)	0.855
V_T_, mL	1206 (391)	1173 (390)	1251 (401)	0.178
Breathing frequency, breaths·min^−1^	33.5 (8.2)	34.0 (7.0)	32.0 (6.1)	0.032
V_D_/V_T_	0.31 (0.08)	0.31 (0.08)	0.31 (0.07)	0.758
PaO_2_, mmHg	62.2 (12.5)	60.1 (11.7)	68.0 (13.7)	< 0.0001
PaCO_2_, mmHg	41.3 (6.7)	42.0 (6.8)	40.3 (6.2)	0.086
ΔPaCO_2_, mmHg	3.9 (4.3)	4.4 (4.3)	2.6 (3.7)	0.003
Plasma lactate, mg·dL^−1^	27.7 (13.4)	26.8 (12.8)	30.6 (15.4)	0.100
Plasma norepinephrine, ng·mL^−1^	2.48 (1.46)	2.45 (1.40)	2.35 (1.48)	0.648
pH	7.365 (0.041)	7.363 (0.039)	7.369 (0.047)	0.324

*Values are presented as mean (SD) and were analyzed using the Student's t-test. BMI, body mass index; ΔPaCO_2_, peak PaCO_2_ – resting PaCO_2_; FEV_1_, forced expiratory volume in 1 s; FVC, forced vital capacity; GOLD I, FEV1% predicted ≥ 80%; II, 80% > FEV1% predicted ≥ 50%; III, 50% > FEV1% predicted ≥ 30%; IV, FEV1% predicted < 30%; HR, heart rate; PaO_2_, arterial oxygen tension; PaCO_2_, arterial carbon dioxide tension; VC, vital capacity; V_D_/V_T_, physiologic dead space/tidal volume ratio; ${\overset{{}^{\circ}}{\text{V}}}_{E}$, minute ventilation; ${\overset{{}^{\circ}}{\text{V}}}_{\text{O2}}$, oxygen uptake; V_T_, tidal volume*.

### Comparison between the dyspnea and leg fatigue groups

In comparison with the dyspnea group, the leg fatigue group showed a significantly lower severity of dyspnea (Borg scale) and breathing frequency at peak exercise and ΔPaCO_2_; significantly higher values of PaO_2_ at peak exercise and breathing reserve; and no significant differences in % predicted FEV_1_, body mass index, V_D_/V_T_, and ${\overset{{}^{\circ}}{\text{V}}}_{\text{O}2}$ at peak exercise (Table 1). Three patients with histories of ischemic heart disease or hypertrophic cardiomyopathy stopped CPET due to leg fatigue.

#### In the dyspnea group

##### Relationships among exercise intolerance, airflow limitation, and breathing pattern

The overall peak ${\overset{{}^{\circ}}{\text{V}}}_{\text{O}2}$ correlated with the FEV_1_ (r^2^ = 0.33, *p* < 0.0001), but the correlation between peak ${\overset{{}^{\circ}}{\text{V}}}_{\text{O}2}$ and FEV_1_ in each subgroup was almost negligible (A: r^2^ = 0.04, *p* = 0.288; B: r^2^ = 0.03, *p* = 0.200; C: r^2^ = 0.00, *p* = 0.802; D: r^2^ = 0.09, *p* = 0.049).

**Figure 3:**
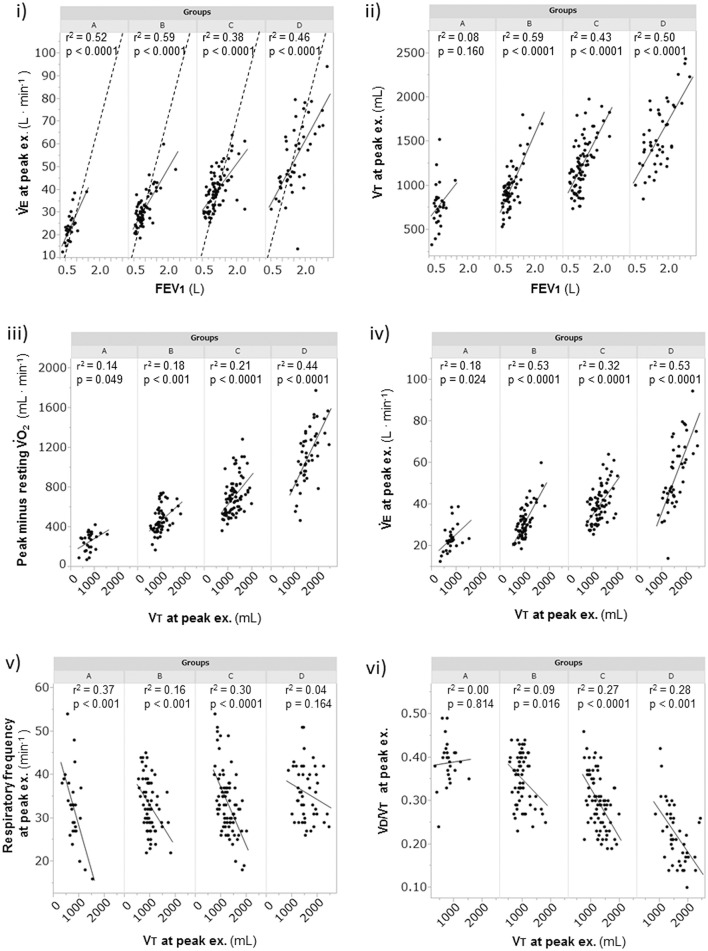
The relationships of the FEV_1_ and the V_T_ at peak exercise during cardiopulmonary exercise testing. (i) relationship of ${\overset{{}^{\circ}}{\text{V}}}_{\text{E}}$ to FEV_1_, (ii) relationship of V_T_ to FEV_1_, (iii) relationship of obtained ${\overset{{}^{\circ}}{\text{V}}}_{O2}$ to V_T_, (iv) relationship of ${\overset{{}^{\circ}}{\text{V}}}_{\text{E}}$ to V_T_, (v) relationship of respiratory frequency to V_T_, and (vi) relationship of V_D_/V_T_ to V_T_. Group A (< 11 mL·min^−1^·kg^−1^); group B (11 to < 15 mL·min^−1^·kg^−1^); group C (15 to < 21 mL·min^−1^·kg^−1^); and group D (≥21 mL·min^−1^·kg^−1^). The dotted lines represent indirect MVV, which was calculated as FEV_1_ × 35. EX., exercise; FEV_1_, forced expiratory volume in 1 s; MVV, maximum voluntary ventilation; V_D_/V_T_, physiologic dead space/tidal volume ratio; ${\overset{{}^{\circ}}{\text{V}}}_{\text{E}}$, minute ventilation; ${\overset{{}^{\circ}}{\text{V}}}_{\text{O}2}$, oxygen uptake; V_T_, tidal volume.

Overall, in the dyspnea subgroups, the FEV_1_ showed good correlations with the ${\overset{{}^{\circ}}{\text{V}}}_{\text{E}}$ (r^2^ = 0.63, *p* < 0.0001) and V_T_ (r^2^ = 0.62, *p* < 0.0001) at peak exercise. Likewise, the FEV_1_ was strongly correlated with the ${\overset{{}^{\circ}}{\text{V}}}_{\text{E}}$ and V_T_ at peak exercise in each subgroup, except with the V_T_ in group A (r^2^ = 0.08) (Figures 3i,ii). Although the breathing reserve was similar among the groups (Table 2), the proportion of patients with ventilatory reserve (more than 10 L/min of breathing reserve) slightly differed among the groups: 2/28 (7%) in group A, 5/64 (8%) in group B, 9/77 (12%) in group C, and 9/46 (20%) in group D. Compared with group D, group A showed significantly lower ${\overset{{}^{\circ}}{\text{V}}}_{\text{E}}$, V_T_, PaO_2_, and plasma lactate level, and significantly higher dead space/tidal volume (V_D_/V_T_) ratio, ${\overset{{}^{\circ}}{\text{V}}}_{\text{E}}$/${\overset{{}^{\circ}}{\text{V}}}_{\text{cO}2}$, PaCO_2_, and bicarbonate ion (${\text{HCO}}_{3}^{-}$) level at peak exercise (Table 2). Among all groups, group A showed the lowest ${\overset{{}^{\circ}}{\text{V}}}_{\text{E}}$ and V_T_ and the highest V_D_/V_T_ ratio at peak exercise. Compared with the results in the other subgroups, the V_T_ at peak exercise in group A was less correlated with the Δ${\overset{{}^{\circ}}{\text{V}}}_{\text{O}2}$ (the peak minus resting ${\overset{{}^{\circ}}{\text{V}}}_{\text{O}2}$) (Figure 3iii) and the ${\overset{{}^{\circ}}{\text{V}}}_{\text{E}}$ (Figure 3iv), was negatively correlated with the respiratory frequency (Figure 3v), and was not correlated with the V_D_/V_T_ ratio (Figure 3vi).

**Table 2:** Baseline characteristics and physiologic findings in the dyspnea group (*N* = 215).

**${\overset{{}^{\circ}}{\text{V}}}_{\text{O2}}$ at peak exercise, mL · min^−1^· kg^−1^**	**A (*n* = 28)**	**B (*n* = 64)**	**C (*n* = 77)**	**D (*n* = 46)**


	9.3 (1.4)	13.0 (1.1)	18.0 (1.8)	25.0 (2.9)
Age, years	71.6 (6.7) ‡‡	71.4 (7.6) ¶¶¶	70.7 (7.1) ##	66.2 (6.1)
BMI, kg·m^−2^	19.6 (2.9)	20.6 (3.1)	20.6 (2.8)	20.8 (3.5)
**PULMONARY FUNCTION TEST**				
FEV_1_, L	0.69 (0.21) ^††††^ ‡‡‡‡	0.91 (0.34) § ¶¶¶¶	1.13 (0.40) ####	1.57 (0.59)
% Predicted FEV_1_, %	29.1 (9.3) ^††††^ ‡‡‡‡	36.1 (11.6) § ¶¶¶¶	43.6 (14.7) ####	57.0 (18.3)
VC, L	2.43 (0.68) ^†^ ‡‡‡‡	2.66 (0.68) ¶¶¶¶	2.92 (0.68) ##	3.36 (0.80)
**CARDIOPULMONARY EXERCISE TESTING**				
At Rest			
Plasma norepinephrine, ng·mL^−1^	0.80 (0.43) ‡‡‡	0.71 (0.31) ¶	0.70 (0.32) #	0.52 (0.18)
At Peak Exercise
Dyspnea, Borg scale	7.7 (2.3)	6.8 (2.6)	7.5 (2.4)	8.0 (1.7)
Dyspnea index, %	100.4 (19.1)	102.9 (22.6)	108.3 (26.1)	107.8 (28.9)
Breathing reserve, L·min^−1^	0.5 (5.2)	0.8 (7.8)	−0.6 (10.9)	−0.2 (13.8)
%HR reserve, %	77.1 (10.6) ^†^ ‡‡	78.2 (15.5) §§ ¶¶¶	85.7 (12.4)	88.7 (12.1)
${\overset{{}^{\circ}}{\text{V}}}_{\text{E}}$, L·min^−1^	23.6 (6.4) ^**^^††††^ ‡‡‡‡	31.2 (7.6) §§§§ ¶¶¶¶	40.0 (8.7) ####	55.1(14.7)
V_T_, mL	780 (245) ^*^^††††^ ‡‡‡‡	981 (253) §§§§ ¶¶¶¶	1245 (295) ####	1561 (372)
Breathing frequency, breaths·min^−1^	32 (8)	33 (6)	34 (7)	36 (6)
${\overset{{}^{\circ}}{\text{V}}}_{\text{E}}$/VCO_2_	54 (12) ^**^^††††^‡‡‡‡	47 (9) § ¶¶¶¶	42 (9) #	37 (9)
V_D_/V_T_	0.39 (0.05) ^††††^ ‡‡‡‡	0.35 (0.06) §§§§ ¶¶¶¶	0.30 (0.06) ####	0.22 (0.07)
PaO_2_, mmHg	53.5 (11.4) ^†^ ‡‡	59.1 (10.6)	61.2 (12.2)	63.6 (11.2)
PaO_2_-slope, mmHg/(mL·min^−1^)^−1^	−7.2 (3.6) ^****^^††††^ ‡‡‡‡	−3.8 (2.5) ¶¶	−3.2 (1.7)	−2.2 (1.9)
PaCO_2_, mmHg	44.2 (7.9) ‡	42.9 (6.9)	41.9 (6.7)	39.7 (5.6)
A-aDo_2_, mmHg	41.0 (10.6)	37.0 (11.8)	36.7 (13.8)	36.5 (12.2)
Plasma lactate, mg·dL^−1^	18.2 (4.9) ^††^ ‡‡‡‡	20.8 (7.4) §§ ¶¶¶¶	27.6 (9.0) ####	40.0 (17.4)
${\text{HCO}}_{3}^{-}$, mEq·L^−1^	25.2 (4.0) ^†^ ‡‡‡‡	24.4 (2.7) ¶¶¶¶	23.3 (2.6) #	21.7 (3.0)
Plasma norepinephrine, ng·mL^−1^	2.35 (1.47)	2.03 (1.17) §	2.72 (1.51)	2.66 (1.38)
Increase in plasma norepinephrine/Δ$\overset{{}^{\circ}}{\text{V}}$O_2_ ng·mL^−2^ · min	7.0 (6.0) ^****^^††††^ ‡‡‡‡	3.0 (2.3)	3.0 (2.1)	2.2 (2.2)
pH	7.370 (0.029)	7.371 (0.043)	7.359 (0.039)	7.355 (0.035)

*Values are presented as mean (SD) and were analyzed using the Tukey–Kramer honestly significant difference test. A-aDo_2_, alveolar-arterial oxygen difference; BMI, body mass index; FEV_1_, forced expiratory volume in one second; ${\text{HCO}}_{3}^{-}$, bicarbonate ion; HR, heart rate; PaO_2_, arterial oxygen tension; PaCO_2_, arterial carbon dioxide tension; VC, vital capacity; ${\overset{{}^{\circ}}{\text{V}}}_{D}$/${\overset{{}^{\circ}}{\text{V}}}_{T}$, physiologic dead space/tidal volume ratio; ${\overset{{}^{\circ}}{\text{V}}}_{E}$, minute ventilation; ${\overset{{}^{\circ}}{\text{V}}}_{T}$, tidal volume; ${\overset{{}^{\circ}}{\text{V}}}_{O2}$, oxygen uptake; ${\overset{{}^{\circ}}{\text{V}}}_{E}$/$\overset{{}^{\circ}}{V}$CO_2_, ventilatory equivalent for carbon dioxide; PaO_2_-slope, peak PaO_2_ – resting PaO_2_/Δ${\overset{{}^{\circ}}{\text{V}}}_{O2}$ (peak ${\overset{{}^{\circ}}{\text{V}}}_{\text{O2}}$ – resting ${\overset{{}^{\circ}}{\text{V}}}_{\text{O2}}$)*.

A vs. B;

* *p < 0.05*,

** *p < 0.01*,

*** *p < 0.001*,

**** *p < 0.0001*.

A vs. C:

† *p < 0.05*,

†† *p < 0.01*,

†††† *p < 0.0001*.

A vs. D:

‡ *p < 0.05*,

‡‡ *p < 0.01*,

‡‡‡ *p < 0.001*,

‡‡‡‡ *p < 0.0001*.

B vs. C:

§ *p < 0.05*,

§§ *p < 0.01*,

§§§§ *p < 0.0001*.

B vs. D:

¶ *p < 0.05*,

¶¶ *p < 0.01*,

¶¶¶ *p < 0.001*,

¶¶¶¶ *p < 0.0001*.

C vs. D:

# *p < 0.05*,

## *p < 0.01*,

#### *p < 0.0001*.

##### Exercise-induced hypoxemia and sympathetic activity level

**Figure 4:**
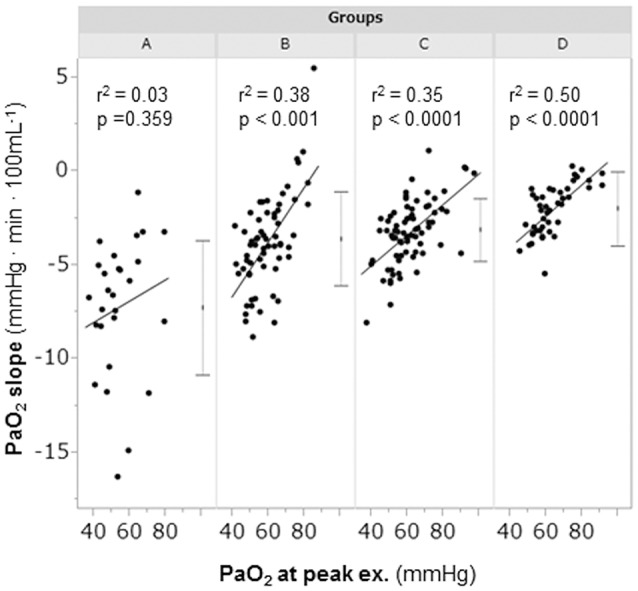
The relationships between the PaO_2_-slope and the PaO_2_ at peak exercise during cardiopulmonary exercise testing. Group A (< 11 mL·min^−1^·kg^−1^); group B (11 to < 15 mL·min^−1^·kg^−1^); group C (15 to < 21 mL·min^−1^·kg^−1^); and group D (≥21 mL·min^−1^·kg^−1^). The horizontal lines represent the standard deviation. The dots between the horizontal lines represent the mean. PaO_2_, arterial oxygen tension.

**Figure 5:**
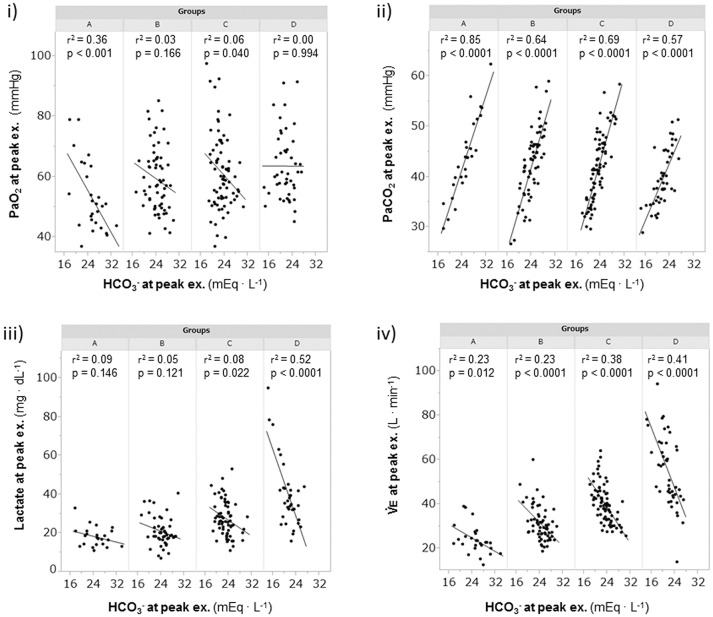
The relationships of the ${\text{HCO}}_{3}^{-}$ levels at peak exercise during cardiopulmonary exercise testin. (i) relationship between PaO_2_ and ${\text{HCO}}_{3}^{-}$, (ii) relationship between PaCO_2_ and ${\text{HCO}}_{3}^{-}$, (iii) relationship between lactate and ${\text{HCO}}_{3}^{-}$, and (iv) relationship between ${\overset{{}^{\circ}}{\text{V}}}_{\text{E}}$ and ${\text{HCO}}_{3}^{-}$. Group A (< 11 mL·min^−1^·kg^−1^); group B (11 to < 15 mL·min^−1^·kg^−1^); group C (15 to < 21 mL·min^−1^·kg^−1^); and group D (≥21 mL·min^−1^·kg^−1^). ${\text{HCO}}_{3}^{-}$, bicarbonate ion; PaCO_2_, arterial carbon dioxide tension; PaO_2_, arterial oxygen tension; ${\overset{{}^{\circ}}{\text{V}}}_{\text{E}}$, minute ventilation.

A considerable number of patients showed exercise-induced hypoxemia (i.e., decrease in PaO_2_ to less than 60 mmHg during exercise) in all the groups: 21/28 (75%) in group A, 37/64 (58%) in group B, 40/77 (52%) in group C, and 21/46 (46%) in group D. In group A, PaO_2_ decreased in almost all patients, although some patients did not show progression to hypoxemia, and the PaO_2_-slope was the steepest among the PaO_2_-slopes in all the groups (A, −7.2 ± 3.6 mmHg·min·100 mL−1; B, −3.8 ± 2.5 mmHg·min·100 mL−1; C, −3.2 ± 1.7 mmHg·min·100 mL−1; D, −2.2 ± 1.9 mmHg·min·100 mL−1; *p* < 0.0001). In group A, the PaO_2_ at peak exercise did not correlate with the PaO_2_-slope (Figure 4), but it negatively correlated with the HCO_3_– level; this correlation was the strongest among all subgroups (Figure 5i). Compared with group D, each subgroup had significantly higher plasma norepinephrine levels at rest (Table 2). In group A, the increases in plasma norepinephrine concentrations during light workload exercise were similar to those in group D during heavy workload exercise.

##### Mechanism of exercise-induced acidosis

**Figure 6:**
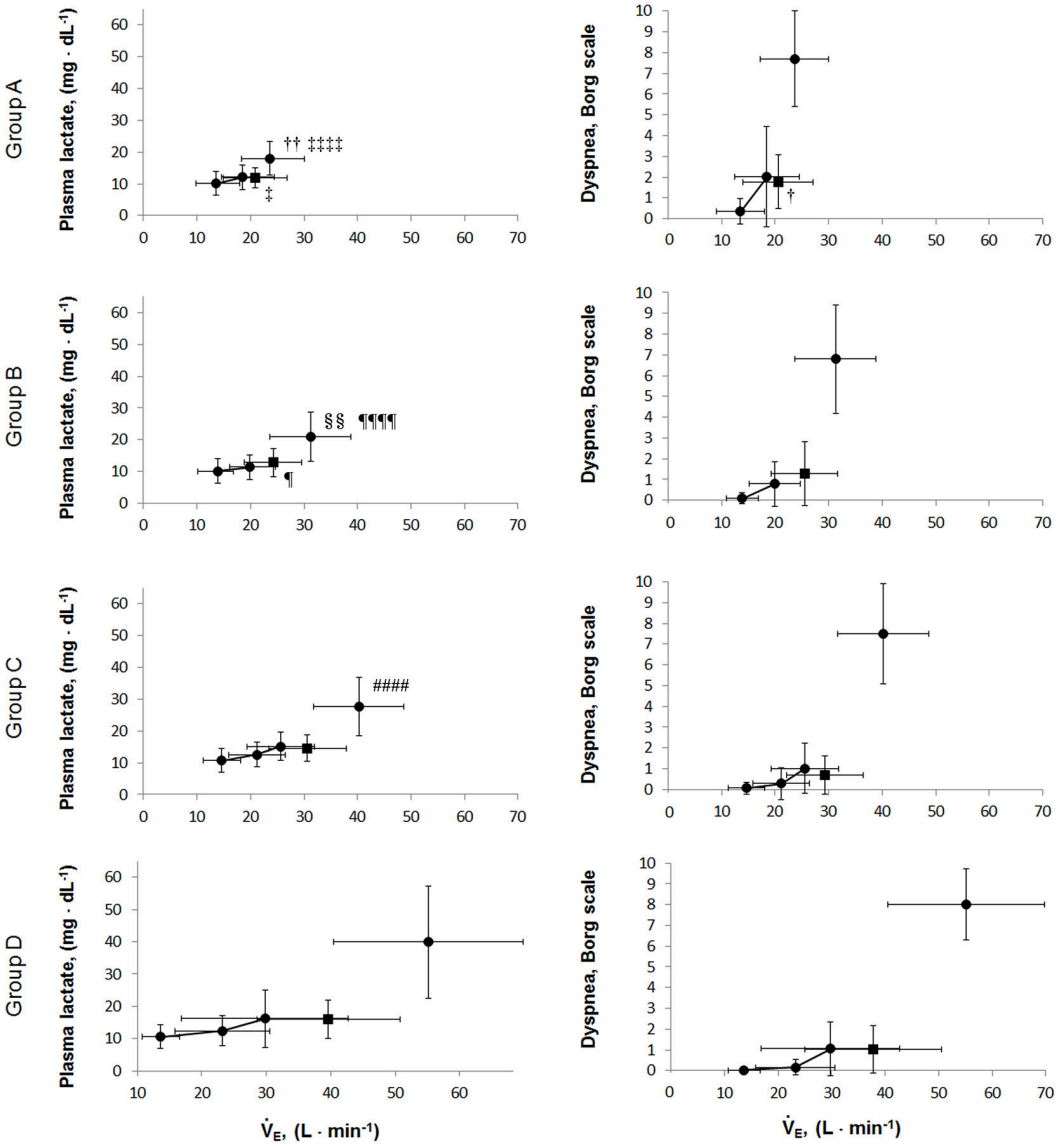
The relationships of the plasma lactate levels and dyspnea borg scales during exercise. Values are presented as mean (SD) and were analyzed using the Tukey-Kramer honestly significant difference test. Circular symbols show exertional variables at rest, during exercise, and at peak exercise. Square symbols show plasma lactate and dyspnea borg scale break points. Plasma lactate borg scale break points were determined in 15/28 in group A, 43/64 in group B, 55/77 in group C, and 42/46 group D. Dyspnea borg scale break points were determined in 14/28 in group A, 46/64 in group B, 62/77 in group C, and 40/46 group D. A vs. B; ^**†**^p < 0.05, ^**††**^p < 0.01, A vs. D: ^**‡**^p < 0.05, ^**‡‡‡‡**^p < 0.0001; B vs. C: ^§§^p < 0.01, B vs. D: ^**¶**^p < 0.05, ^**¶¶¶¶**^p < 0.0001; C vs. D: ^####^p < 0.0001.

The relationships of ${\text{HCO}}_{3}^{-}$ levels with PaCO_2_, plasma lactate levels, and ${\overset{{}^{\circ}}{\text{V}}}_{\text{E}}$ at peak exercise are shown in Figure 5. At peak exercise, the ${\text{HCO}}_{3}^{-}$ level was significantly correlated with the PaCO_2_ and ${\overset{{}^{\circ}}{\text{V}}}_{\text{E}}$ in all groups, but it did not correlate with the plasma lactate level in groups A and B (Figures 5ii–iv). The V_T_-break points were determined in 17 of the 28 (61%) patients in group A. In the patients with V_T_-break points in group A, there was a significant negative correlation between the ${\text{HCO}}_{3}^{-}$ level at peak exercise and the V_T_ level corresponding to the V_T_-break point (r = −0.52, *p* = 0.032). This suggested that when there was severe hyperinflation, respiratory acidosis, rather than lactic acidosis, primarily occurred. The proportion of patients who maintained an ${\text{HCO}}_{3}^{-}$ level of more than 24 mEq·L^−1^ at peak exercise was highest in group A (19/28, 68%), in comparison with group B (38/64, 59%), group C (24/77, 31%), and group D (12/46, 26%). However, there were no significant differences in the pH levels and Borg scales at peak exercise among the subgroups (Table 2, Figure 6). Although the plasma lactate level and ${\overset{{}^{\circ}}{\text{V}}}_{\text{E}}$ at peak exercise showed lower values in the group with decreased exercise tolerance (Table 2, Figure 6), the PaO_2_ and PaCO_2_ values at peak exercise varied widely among the individual patients in all groups.

#### In the other EL group

Ten (3.6%) of 280 COPD patients who had no comorbidities stopped exercise due to ECG changes (Figure 2).

## Discussion

This study evaluated the pathophysiologic conditions related to exertional dyspnea among COPD patients using CPET with arterial blood analysis in clinical practice. The exertional conditions during exercise differed according to the residual exercise performance. Moreover, the COPD patients also showed great inter-individual variability. The appropriate expansion of ventilation caused by an increased ventilatory drive due to the arterial blood acidosis (metabolic and/or respiratory acidosis) and hypoxemia is constricted in the setting of reduced respiratory system ability (reduced ventilatory capacity). These conditions provoke exertional dyspnea.

### Pathophysiologic conditions in the leg fatigue group

Compared with the dyspnea group, the leg fatigue group demonstrated a lower severity of dyspnea and PaCO_2_ retention and a higher PaO_2_ level at peak exercise with similar peak ${\overset{{}^{\circ}}{\text{V}}}_{\text{O}2}$. The breathing reserve has been considered to influence breathing discomfort and alveolar gas exchange. Given that the present study showed that the cardinal symptoms for exercise intolerance were dyspnea and/or fatigue, which may result from one or a combination of limitations, such as ventilatory constraints, pulmonary gas exchange abnormalities, peripheral muscle dysfunction, and cardiac dysfunction, evaluation of the exercise-limiting factor in each case is fundamental for developing treatment strategies for COPD (Laveneziana et al., 2007; Cote et al., 2008; Watz et al., 2014). In the present study, 20% (*n* = 58) of the patients who stopped CPET due to leg fatigue showed better functional ventilatory capacity than the dyspnea group. One recent report suggested that COPD patients who developed leg fatigue during exercise training had greater functional exercise tolerance than those who do not (Burtin et al., 2012). These results implied that pulmonary rehabilitation (exercise training) should be indicated for COPD patients whose exercise intolerance is related to leg fatigue.

### Relationship of exercise intolerance with airflow limitation and breathing pattern

Exercise tolerance, which can be measured by the peak ${\overset{{}^{\circ}}{\text{V}}}_{\text{O}2}$, depends on the intensity and duration of exercise (Neder et al., 2000; Oga et al., 2005). Similar to other reports (Cahalin et al., 1995; Bauerle et al., 1998; Foglio et al., 2000; Agusti et al., 2010), the present study showed that prediction of exercise tolerance using the peak ${\overset{{}^{\circ}}{\text{V}}}_{\text{O}2}$ level based on the FEV_1_ was a challenge in all groups, except in group D of the dyspnea group, which showed the highest exercise tolerance, although the FEV_1_ has been indispensable for COPD diagnosis and might be a parameter useful for assessment of the effects of inhaled medications. In other words, increasing the FEV_1_ alone might not help increase exercise tolerance in COPD patients. Oga et al. (2005) reported that evaluation of the peak ${\overset{{}^{\circ}}{\text{V}}}_{\text{O}2}$, rather than the FEV_1_, over time was more helpful in understanding the changes in a patient's condition. Macklem (2010) reported that tachypnea in a COPD patient decreases dynamic lung compliance and that rapid shallow breathing is generally considered to cause difficulty in breathing. In group A, based on the negligible correlation of the Δ${\overset{{}^{\circ}}{\text{V}}}_{\text{O}2}$ with a reduced V_T_, which has a negative correlation with respiratory frequency at peak exercise (Figures 3iii,v), and the absence of correlation between V_T_ and the V_D_/V_T_ ratio, there were basically two breathing patterns during exercise, i.e., a relatively stable increase of V_T_ with low respiratory frequency (slow exhalation breathing) and a reduced increase of V_T_ with high respiratory frequency (rapid shallow breathing). Of note, regardless of the two breathing patterns, group A showed a low exercise tolerance due to wasted ventilation. These imply that care for patients with low exercise tolerance should be especially individualized.

### Relationship between exercise-induced hypoxemia and sympathetic activity level

In COPD, evaluation of the degree of exercise-induced hypoxemia (PaO_2_-slope) along with assessment of exercise tolerance is important for predicting survival and understanding the varying pathophysiologic conditions among individual patients (Hiraga et al., 2003; Maekura et al., 2014, 2015; Yoshimura et al., 2014). Recently, the members of the Long-Term Oxygen Treatment Trial Research Group reported that long-term supplemental oxygen therapy had no significant effect on mortality among stable COPD patients with either resting or exercise-induced moderate hypoxemia, as measured by pulse oximetry, during a 6-min walk distance test (Albert et al., 2016). However, in that study, the oxygen saturation (SpO_2_) measurements were obtained in individuals with different workloads. The severity of pulmonary gas exchange abnormalities should be evaluated by a decrease in PaO_2_ over a fixed workload or by an increase in oxygen uptake (PaO_2_-slope). Moreover, the use of SpO_2_ during a 6-min walk test to evaluate the severity of exercise-induced hypoxemia will produce inaccurate results. In the present study, the findings clearly showed that PaO_2_ decreased to less than 60 mmHg in half of the patients, even in group C and D patients with exercise tolerance. Furthermore, 25% of the patients in group A did not show hypoxemia at peak exercise. However, the PaO_2_ slope became steep with decreasing exercise tolerance, especially in group A, in which the PaO_2_-slope did not correlate with the PaO_2_ at peak exercise; that is, the PaO_2_-slope had a greater impact on exercise intolerance (reduced Δ${\overset{{}^{\circ}}{\text{V}}}_{\text{O}2}$) in group A than in the other subgroups (Figure 4). In addition, at peak exercise, the PaO_2_ most negatively correlated with the ${\text{HCO}}_{3}^{-}$ level in group A (Figure 5i); that is, a severe degree of exercise-induced hypoxemia occurred with reduced exercise tolerance to reach respiratory acidosis. This might explain the elevated sympathetic activity, which manifested as a 2-fold increase in the plasma norepinephrine level in group A already from the resting condition (Maekura et al., 2014; Yoshimura et al., 2014). Their findings might affect the heterogeneity of the pathophysiologic conditions underlying COPD during exercise. A prolonged increase in sympathetic activity can cause weight loss, anxiety, and other stress-related body changes, including hypoxemia and exertional acidosis. For example, reduced digestive activity results from a generally inhibitory effect of norepinephrine on the enteric nervous system by decreasing gastrointestinal motility, blood flow, and secretion of digestive substances (Konturek et al., 2004). Furthermore, the PaO_2_-slope, rather than the PaO_2_ or SpO_2_ value at the end of exercise, was the factor that affected the prognosis of COPD (Hiraga et al., 2003; Maekura et al., 2014; Yoshimura et al., 2014). Therefore, the focus should be on the PaO_2_-slope for individualized patient care, in order to improve or avoid severe and life-threatening exercise-induced hypoxemia (Maekura et al., 2015).

### Mechanism of exercise-induced acidosis

The varying ventilation to compensate for exercise-induced acidosis in COPD might be related with dynamic hyperinflation (O'Donnell et al., 2009; Wasserman et al., 2014), which is considered a key contributor to dyspnea. In group A, the main cause of exercise intolerance might be the wasted ventilation from varying breathing patterns, including slow exhalation breathing and rapid shallow breathing, as shown in Figure 3v. In the present study, exertional acidosis was caused by both metabolic and respiratory factors to stop exercise in each group (Figure 5). Furthermore, in subgroup A of the dyspnea group, in which dynamic hyperinflation was especially confirmed using V_T_-break points, exercise was stopped primarily because of respiratory acidosis, that is, these patients did not develop ventilatory compensation in response to respiratory acidosis; therefore, the ${\text{HCO}}_{3}^{-}$ level was high at peak exercise (Table 2). Evaluating ${\text{HCO}}_{3}^{-}$ could further the understanding of the pathophysiologic conditions, by elucidating the state of acidosis conditions when inadequate ventilatory compensation including hyperinflation occurs during exercise. On the other hand, compared with group A patients, group D patients in the dyspnea group had a lower V_D_/V_T_ ratio and were able to maintain ventilation capacity; moreover, in that group, there was a negative correlation of the ${\text{HCO}}_{3}^{-}$ level with ventilatory capacity and plasma lactate levels (Table 2, Figure 5). In addition, the proportion of patients who maintained an ${\text{HCO}}_{3}^{-}$ level greater than 24 mEq · L^−1^ at peak exercise was lower in group D (26%) than in group A (68%). Taken together, the group D patients with higher ventilatory capacity stopped exercise primarily because of lactic acidosis (Figure 6). It is interesting to note that in this study, we assumed that cessation of exercise in these patients was due to their inability to compensate for the exertional acidosis and that there were no significant differences in the pH levels and Borg scales at peak exercise in all subgroups in the dyspnea group (Table 2, Figure 6). Based on our previous CPET evaluations using two kinds of inhaled oxygen concentrations in patients with COPD and even idiopathic pulmonary fibrosis, exercise limitation often depended on limitations in ventilatory compensation for exertional acidosis, rather than hypoxemia during exercise (Miki et al., 2012, 2013). These findings suggest that although the degree of exertional hypoxemia and the breathing pattern vary in COPD patients, pH homeostasis during exercise might be regulated by ventilation, which might be related to dynamic hyperinflation.

This study had some limitations. First, all patients in this study were grouped according to peak ${\overset{{}^{\circ}}{\text{V}}}_{\text{O}2}$ increments obtained by CPET and combined with those showing a similar degree of FEV_1_. The relationship between FEV1 and peak ${\overset{{}^{\circ}}{\text{V}}}_{\text{O}2}$ might vary among the studies, given that the severity of airflow limitation differs with respect to each study population. Although COPD is characterized by airflow limitation due to airway and/or alveolar abnormality, the chief complaint is exercise intolerance due to breathlessness. The peak ${\overset{{}^{\circ}}{\text{V}}}_{\text{O}2}$ in incremental exercise is also the gold standard of exercise capacity.

Second, the patients were asked to make their maximal efforts before the CPET, which was performed until patient exhaustion. Certainly, the level of exhaustion to stop exercise might have varied among patients and affected the results. However, arterial blood parameters, including the PaO_2_-slope, are completely objective valuables. Moreover, the applicability of our results to the entire spectrum of COPD patients remains to be investigated.

## Conclusion

Our findings demonstrated the heterogeneity of the pathophysiologic conditions underlying COPD during exercise, even when the dyspnea level was similar. Ventilation is stimulated to avoid exertional acidosis and hypoxemia; however, the responses are limited by the ventilatory impairment in COPD patients. This ventilatory decompensation might be one of the common mechanisms contributing exertional dyspnea. The resting variables (FEV_1_) could not accurately predict exercise tolerance and pathophysiology during exercise in COPD patients. Therefore, CPET should be implemented before initiating interventions such as pulmonary rehabilitation in these patients. Understanding the individual exercise limitations of COPD patients could provide helpful information on a suitable management strategy for the patients.

## Data availability statement

This study does not include any clinical dataset to be shared. The datasets used and/or analyzed during the current study are available from the corresponding author on reasonable request.

## Author contributions

All authors contributed to the conceptualization and design of the study. HK, KM, and RM contributed to data abstraction and analysis. HK drafted the initial manuscript. All authors contributed to manuscript writing and approved the submission of the final manuscript. KM and RM are the guarantors of this work.

### Conflict of interest statement

The authors declare that the research was conducted in the absence of any commercial or financial relationships that could be construed as a potential conflict of interest.

## Abbreviations

COPD: chronic obstructive pulmonary disease
CPET: cardiopulmonary exercise testing
ECG: electrocardiogram
EL: exercise limitations
FEV_1_: forced expiratory volume in one second
${\text{HCO}}_{3}^{-}$: bicarbonate ion
HR: heart rate
NE: plasma norepinephrine level
PaCO_2_: arterial carbon dioxide tension
PaO_2_: arterial oxygen tension
${\overset{{}^{\circ}}{\text{V}}}_{\text{CO}2}$: carbon dioxide output
V_D_/V_T_: physiologic dead space/tidal volume ratio
${\overset{{}^{\circ}}{\text{V}}}_{\text{E}}$: minute ventilation
${\overset{{}^{\circ}}{\text{V}}}_{\text{O}2}$: oxygen uptake
V_T_: tidal volume.

## References

[B1] Agusti A. Calverley P. M. Celli B. Coxson H. O. Edwards L. D. Lomas D. A.. (2010). Characterisation of COPD heterogeneity in the ECLIPSE cohort. Resp. Res. 11:122. 10.1186/1465-9921-11-122 20831787 PMC2944278

[B2] Albert R. K. Au D. H. Blackford A. L. Casaburi R. Cooper J. A. Jr. Criner G. J.. (2016). A randomized trial of long-term oxygen for COPD with moderate desaturation. N. Engl. J. Med. 375, 1617–1627. 10.1056/NEJMoa1604344 27783918 PMC5216457

[B3] American Thoracic Society (1995a). Standards for the diagnosis and care of patients with chronic obstructive pulmonary disease. Am. J. Respir. Crit. Care Med. 152, S77–S121. 7582322

[B4] American Thoracic Society (1995b). Standardization of spirometry, 1994 update. Am. J. Respir. Crit. Care Med. 152, 1107–1136. 10.1164/ajrccm.152.3.7663792 7663792

[B5] Bauerle O. Chrusch C. A. Younes M. (1998). Mechanisms by which COPD affects exercise tolerance. Am. J. Resp. Crit. Care Med. 157, 57–68. 10.1164/ajrccm.157.1.9609126 9445279

[B6] Borg G. A. (1982). Psychophysical bases of perceived exertion. Med. Sci. Sports Exerc. 14, 377–381. 10.1249/00005768-198205000-00012 7154893

[B7] Borrill Z. L. Houghton C. M. Woodcock A. A. Vestbo J. Singh D. (2005). Measuring bronchodilation in COPD clinical trials. Br. J. Clin. Pharmacol. 59, 379–384. 10.1111/j.1365-2125.2004.02261.x 15801931 PMC1884799

[B8] Burtin C. Saey D. Saglam M. Langer D. Gosselink R. Janssens W.. (2012). Effectiveness of exercise training in patients with COPD: the role of muscle fatigue. Eur. Resp. J. 40, 338–344. 10.1183/09031936.00111811 22135284

[B9] Cahalin L. Pappagianopoulos P. Prevost S. Wain J. Ginns L. (1995). The relationship of the 6-min walk test to maximal oxygen consumption in transplant candidates with end-stage lung disease. Chest 108, 452–459. 10.1378/chest.108.2.452 7634883

[B10] Clinical exercise testing with reference to lung diseases: indications standardization and interpretation strategies (1997). ERS task force on standardization of clinical exercise testing. European respiratory society. Eur. Resp. J. 10, 2662–2689. 10.1183/09031936.97.10112662 9426113

[B11] Cote C. G. Pinto-Plata V. M. Marin J. M. Nekach H. Dordelly L. J. Celli B. R. (2008). The modified BODE index: validation with mortality in COPD. Eur. Resp. J. 32, 1269–1274. 10.1183/09031936.00138507 18579541

[B12] Foglio K. Carone M. Pagani M. Bianchi L. Jones P. W. Ambrosino N. (2000). Physiological and symptom determinants of exercise performance in patients with chronic airway obstruction. Resp. Med. 94, 256–263. 10.1053/rmed.1999.0734 10783937

[B13] Global Initiative for Chronic Obstructive Lung Disease. GOLD (2017). Global Strategy for the Diagnosis, Management and Prevention of COPD. Available online at: http://goldcopd.org/gold-2017-global-strategy-diagnosis-management-prevention-copd/. (Accessed May 12, 2017).

[B14] Hallstrand T. S. Bates P. W. Schoene R. B. (2000). Aerobic conditioning in mild asthma decreases the hyperpnea of exercise and improves exercise and ventilatory capacity. Chest 118, 1460–1469. 10.1378/chest.118.5.1460 11083702

[B15] Hiraga T. Maekura R. Okuda Y. Okamoto T. Hirotani A. Kitada S.. (2003). Prognostic predictors for survival in patients with COPD using cardiopulmonary exercise testing. Clin. Physiol. Funct. Imaging 23, 324–331. 10.1046/j.1475-0961.2003.00514.x 14617262

[B16] Johnson B. D. Weisman I. M. Zeballos R. J. Beck K. C. (1999). Emerging concepts in the evaluation of ventilatory limitation during exercise: the exercise tidal flow-volume loop. Chest 116, 488–503. 10.1378/chest.116.2.488 10453881

[B17] Konturek S. J. Konturek J. W. Pawlik T. Brzozowski T. (2004). Brain-gut axis and its role in the control of food intake. J. Physiol. Pharmacol. 55, 137–54. 15082874

[B18] Laveneziana P. Palange P. (2012). Physical activity, nutritional status and systemic inflammation in COPD. Eur Resp. J. 40, 522–529. 10.1183/09031936.00041212 22941542

[B19] Laveneziana P. Parker C. M. O'Donnell D. E. (2007). Ventilatory constraints and dyspnea during exercise in chronic obstructive pulmonary disease. App. Physiol. Nutr Metab. 32, 1225–1238. 10.1139/H07-119 18059601

[B20] Macklem P. T. (2010). Therapeutic implications of the pathophysiology of COPD. Eur. Resp. J. 35, 676–80. 10.1183/09031936.00120609 20190332

[B21] Maekura R. Hiraga T. Miki K. Kitada S. Miki M. Yoshimura K.. (2015). Personalized pulmonary rehabilitation and occupational therapy based on cardiopulmonary exercise testing for patients with advanced chronic obstructive pulmonary disease. Int. J. Chron. Obstruct. Pulmon Dis. 10, 1787–1800. 10.2147/COPD.S86455 26366071 PMC4562755

[B22] Maekura R. Hiraga T. Miki K. Kitada S. Yoshimura K. Miki M.. (2014). Differences in physiological response to exercise in patients with different COPD severity. Respir. Care 59, 252–262. 10.4187/respcare.02201 23821762

[B23] Miki K. Maekura R. Hiraga T. Hashimoto H. Kitada S. Miki M.. (2009). Acidosis and raised norepinephrine levels are associated with exercise dyspnoea in idiopathic pulmonary fibrosis. Respirology 14, 1020–1026. 10.1111/j.1440-1843.2009.01607.x 19740262

[B24] Miki K. Maekura R. Hiraga T. Kitada S. Miki M. Yoshimura K.. (2012). Effects of oxygen on exertional dyspnoea and exercise performance in patients with chronic obstructive pulmonary disease. Respirology 17, 149–154. 10.1111/j.1440-1843.2011.02086.x 22008208

[B25] Miki K. Maekura R. Miki M. Kitada S. Yoshimura K. Tateishi Y.. (2013). Exertional acidotic responses in idiopathic pulmonary fibrosis: the mechanisms of exertional dyspnea. Resp. Physiol. Neurobiol. 185, 653–658. 10.1016/j.resp.2012.11.008 23246672

[B26] Neder J. A. Jones P. W. Nery L. E. Whipp B. J. (2000). Determinants of the exercise endurance capacity in patients with chronic obstructive pulmonary disease. the power-duration relationship. Am. J. Resp. Crit. Care Med. 162, 497–504. 10.1164/ajrccm.162.2.9907122 10934077

[B27] O'Donnell D. E. Ora J. Webb K. A. Laveneziana P. Jensen D. (2009). Mechanisms of activity-related dyspnea in pulmonary diseases. Resp. Physiol. Neurobiol. 167, 116–132. 10.1016/j.resp.2009.01.010 19450767

[B28] Oga T. Nishimura K. Tsukino M. Sato S. Hajiro T. Mishima M. (2005). Exercise capacity deterioration in patients with COPD: longitudinal evaluation over 5 years. Chest 128, 62–69. 10.1378/chest.128.1.62 16002917

[B29] Palange P. Ward S. A. Carlsen K. H. Casaburi R. Gallagher C. G. Gosselink R.. (2007). Recommendations on the use of exercise testing in clinical practice. Eur. Resp. J. 29, 185–209. 10.1183/09031936.00046906 17197484

[B30] Parshall M. B. Schwartzstein R. M. Adams L. Banzett R. B. Manning H. L. Bourbeau J.. (2012). An official American Thoracic Society statement: update on the mechanisms, assessment, and management of dyspnea. Am. J. Resp. Crit. Care Med. 185, 435–452. 10.1164/rccm.201111-2042ST 22336677 PMC5448624

[B31] Pauwels R. A. Buist A. S. Calverley P. M. Jenkins C. R. Hurd S. S. (2001). Global strategy for the diagnosis, management, and prevention of chronic obstructive pulmonary disease: NHLBI/WHO Global Initiative for Chronic Obstructive Lung Disease (GOLD) workshop summary. Am. J. Respir. Crit. Care Med. 163, 1256–1212. 10.1164/ajrccm.163.5.2101039 11316667

[B32] Wasserman K. Cox T. A. Sietsema K. E. (2014). Ventilatory regulation of arterial H(+) (pH) during exercise. Resp. Physiol. Neurobiol. 190, 142–148. 10.1016/j.resp.2013.10.009 24369924

[B33] Wasserman K. H. J. Sue D. Y. Stringer W. W. Whipp B. J. (2005). Principles of Exercise Testing and Interpretation. 4th Edn Philadelphia, PA: Lippincott Williams and Wilkins.

[B34] Watz H. Pitta F. Rochester C. L. Garcia-Aymerich J. ZuWallack R. Troosters T.. (2014). An official European Respiratory Society statement on physical activity in COPD. Eur. Resp. J. 44, 1521–1537. 10.1183/09031936.00046814 25359358

[B35] Yoshimura K. Maekura R. Hiraga T. Miki K. Kitada S. Miki M.. (2014). Identification of three exercise-induced mortality risk factors in patients with COPD. COPD 11, 615–626. 10.3109/15412555.2014.898038 24914923

